# Phosphorus‐Doped Graphene Aerogel as Self‐Supported Electrocatalyst for CO_2_‐to‐Ethanol Conversion

**DOI:** 10.1002/advs.202202006

**Published:** 2022-07-12

**Authors:** Fangqi Yang, Caihong Liang, Haoming Yu, Zheling Zeng, Yeng Ming Lam, Shuguang Deng, Jun Wang

**Affiliations:** ^1^ School of Resource and Environment Nanchang University No. 999 Xuefu Avenue Jiangxi 330031 P. R. China; ^2^ Chemistry and Chemical Engineering School Nanchang University No. 999 Xuefu Avenue Jiangxi 330031 P. R. China; ^3^ Department of Chemistry National University of Singapore 3 Science Drive 3 Singapore 117543 Singapore; ^4^ School of Materials Science and Engineering Nanyang Technological University Singapore 639798 Singapore; ^5^ Facility for Analysis Characterization Testing and Simulation (FACTS) Nanyang Technological University Singapore 639798 Singapore; ^6^ School for Engineering of Matter Transport and Energy Arizona State University 551 E. Tyler Mall Tempe AZ 85287 USA

**Keywords:** CO_2_ reduction, electrocatalysis, ethanol, graphene aerogel, phosphorus

## Abstract

Electrochemical reduction of carbon dioxide (CO_2_) to ethanol is a promising strategy for global warming mitigation and resource utilization. However, due to the intricacy of C─C coupling and multiple proton–electron transfers, CO_2_‐to‐ethanol conversion remains a great challenge with low activity and selectivity. Herein, it is reported a P‐doped graphene aerogel as a self‐supporting electrocatalyst for CO_2_ reduction to ethanol. High ethanol Faradaic efficiency (FE) of 48.7% and long stability of 70 h are achieved at −0.8 V_RHE_. Meanwhile, an outstanding ethanol yield of 14.62 µmol h^−1^ cm^−2^ can be obtained, outperforming most reported electrocatalysts. In situ Raman spectra indicate the important role of adsorbed *CO intermediates in CO_2_‐to‐ethanol conversion. Furthermore, the possible active sites and optimal pathway for ethanol formation are revealed by density functional theory calculations. The graphene zigzag edges with P doping enhance the adsorption of *CO intermediate and increase the coverage of *CO on the catalyst surface, which facilitates the *CO dimerization and boosts the EtOH formation. In addition, the hierarchical pore structure of P‐doped graphene aerogels exposes abundant active sites and facilitates mass/charge transfer. This work provides inventive insight into designing metal‐free catalysts for liquid products from CO_2_ electroreduction.

## Graphical abstract

P‐doped graphene aerogel is used as self‐supporting electrodes for favorable ethanol production from carbon dioxide (CO_2_) electroreduction with ethanol Faradaic efficiency (FE) and yield. This work can inspire the development of metal‐free electrocatalysts for C_2+_ production.

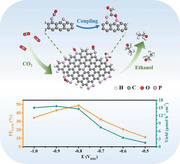

## 1 Introduction

The conversion of carbon dioxide (CO_2_) via renewable but intermittent electricity (e.g., from solar and wind) is regarded as a promising approach to close the anthropogenic carbon cycle and provide value‐added chemical feedstocks.^[^ ^1^, ^2^, ^3^ ^]^ Electrochemical CO_2_ reduction reaction (CO_2_RR) has been intensively investigated (Table S1, Supporting Information), and mostly, tends to selectively form 2‐electron‐transfer products, i.e., carbon monoxide (CO) and formic acid (HCOOH).^[^ ^4^, ^5^ ^]^ Multicarbon (C_2+_) products, particularly ethanol (C_2_H_5_OH, EtOH), are more appreciated due to the higher energy density (26.8 MJ kg^−1^) and ease of storage as liquid fuels.^[^ ^6^, ^7^, ^8^ ^]^ However, the delicate design of efficient CO_2_RR catalysts to selectively generate EtOH via C─C coupling is extremely challenging owing to the multiple proton‐coupled electron transfer processes.^[^ ^9^, ^10^ ^]^ Generally, Cu‐based electrocatalysts are extensively investigated for C_2+_ products, but the product selectivity and Faradic efficiency (FE) for EtOH are still unsatisfactory.^[^ ^7^, ^11^ ^]^ Despite intensive research efforts have been devoted, such as surface property modification by doping/alloying,^[^ ^9^, ^12^ ^]^ grain boundary,^[^ ^13^ ^]^ and in situ reconstruction,^[^ ^14^, ^15^ ^]^ the primary product is still ethylene (C_2_H_4_).

Metal‐free carbon‐based catalysts have shown attractive prospects in CO_2_RR, achieving comparable catalytic activities to metal‐based electrocatalysts while possessing advantages, such as cost‐effectiveness and superior durability.^[^ ^16^, ^17^ ^]^ Heteroatoms doping strategy is adapted to tune the charge densities of active sites by breaking the intrinsic electro‐neutrality of pristine inert carbon matrix.^[^ ^10^, ^18^ ^]^ Nevertheless, nitrogen‐doping (N‐doping) has been proven to be a feasible strategy for boosting CO_2_RR, but the products are mainly limited to CO.^[^ ^17^, ^19^, ^20^ ^]^ During the conversion of CO_2_ to EtOH, various intermediates and transition states are involved, which will significantly alter the activity and selectivity.^[^ ^2^, ^11^ ^]^ The adsorption of *CO intermediate has been widely acknowledged for the C─C coupling.^[^ ^1^, ^21^ ^]^ Whereas, the facile desorption of *CO on conventional carbon electrocatalysts results in difficulties for the formation of C_2+_ products.^[^ ^19^, ^22^ ^]^ Therefore, tailing binding energy and strength of *CO with electrocatalysts through tuning the local electron structure of catalytic sites is critical and essential.

Besides, theoretical studies have demonstrated that the high spin density of N atoms also favors the competing hydrogen evolution reaction (HER).^[^ ^23^ ^]^ To the best of our knowledge, efficient carbon electrocatalysts that can stabilize C1 intermediates for further C─C coupling are rarely reported. For example, Sun et al. have developed the N‐doped ordered mesoporous carbon (c‐NC), which displayed a high EtOH FE of 77% at −0.56 V_RHE_ but with a low production rate.^[^ ^22^ ^]^ Comparably, phosphorus (P) atom has the same number of valence electrons as N atom, which can also enhance the electronic conductivity of carbon substrates. Because the electronegativity of C atom (2.55) lies between P atom (2.19) and N atom (3.04), the polarity of C─P bond is opposite to that of C─N bond. The partial positive‐charged P atoms could serve as the catalytic sites, in contrast to the possible C active sites in the N‐doped carbon matrix. Meanwhile, the larger atomic radius of P (110 pm) than that of C (77 pm) could generate a high distortion in the carbon skeleton and form abundant edge sites.^[^ ^23^, ^24^ ^]^ Thus, we anticipate that the incorporation of P heteroatoms into the carbon matrix may pave a new avenue for efficient EtOH generation.

Herein, we first report P‐doped graphene aerogels (PGAs) as self‐supporting catalysts to facilitate C─C coupling for CO_2_ reduction to EtOH. A high EtOH Faradaic efficiency of 48.7% was attained as well as 70 h stability at −0.8 V_RHE_. Moreover, the EtOH yield reached 14.62 µmol h^−1^ cm^−2^, far outperforming previously reported state‐of‐the‐art electrocatalysts.^[^ ^4^, ^22^, ^25^, ^26^, ^27^ ^]^ In situ Raman spectra identified the key role of *CO intermediate during the CO_2_‐to‐EtOH conversion. Furthermore, density functional theory (DFT) calculations demonstrated that P‐embedded graphene with abundant zigzag edges could significantly reduce the energy barrier for *CO dimerization and thus boost the EtOH formation.

## 2 Results and Discussion

### 2.1 Characterization of the Electrocatalysts

**Figure 1:**
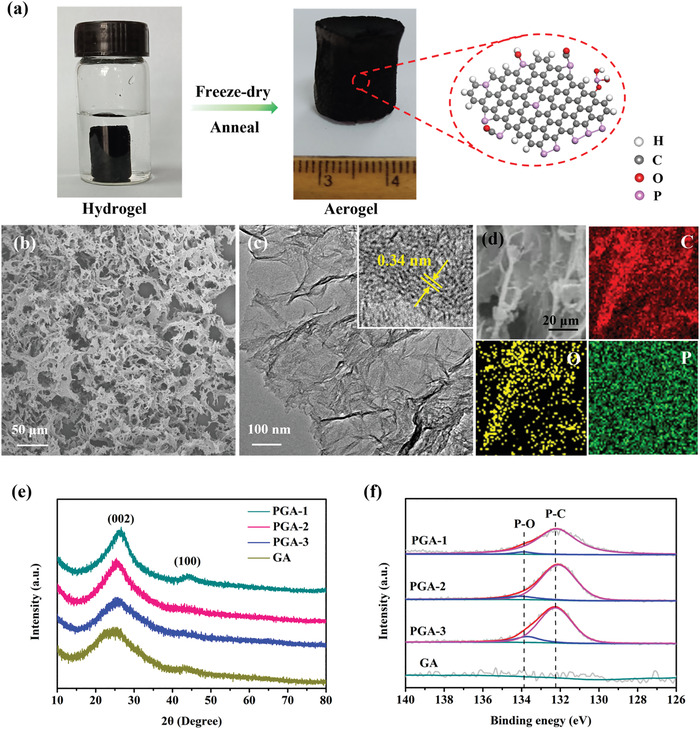
a) Schematic illustration of the synthesis process. b) SEM image, c) TEM image (inset HR‐TEM image), and d) EDS mapping of PGA‐2. e) XRD patterns and f) high‐resolution XPS spectra of P 2p for all samples.

P‐doped graphene hydrogels were prepared by a hydrothermal reduction of dispersed graphene oxide (GO) aqueous solution with varying amounts of phosphoric acid (Figure S1, Supporting Information). During the process, P atoms were incorporated into the graphene sheets simultaneously as the sheets aggregated into 3D composite architecture.^[^ ^28^ ^]^ Thereafter, the hydrogels were dried via freeze‐drying to maintain the monolithic structure and then subjected to thermal treatment to obtain the free‐standing PGAs with 3D hierarchical porous architecture (**Figure** **1**a). Scanning electron microscopy (SEM) images of PGAs showed the interconnected frameworks composed of graphene nanosheets (Figure 1b; and Figure S2a–c, Supporting Information), amongst them, PGA‐2 exhibited an obvious porous architecture. Transmission electron microscopy (TEM) manifested the typical layer‐connected and sheet‐like graphene structures of PGAs (Figure 1c; and Figure S2d–f, Supporting Information). High‐resolution TEM (HR‐TEM) revealed that the few‐layer sheet structure with a lattice spacing of ≈0.34 nm on PGA‐2 (Figure 1c inset), corresponding to the typical *c*‐axis layer‐to‐layer distance of graphite.^[^ ^18^ ^]^ Moreover, the energy‐dispersive X‐ray spectroscopy (EDS) elemental maps demonstrated the uniform distribution of P elements throughout the graphene carbon skeleton (Figure 1d).

The crystalline and defect information were investigated by X‐ray diffraction (XRD) and Raman characterizations. XRD patterns showed two characteristic diffraction peaks at 24.5° and 43.3° for all samples, which can be assigned to the (002) and (100) plane of amorphous graphitic carbon (Figure 1e).^[^ ^19^ ^]^ Raman spectra showed a well‐defined D‐band for defective *sp* ^3^ hybridized carbon and G‐band for *sp* ^2^ hybridized carbon at 1347 and 1589 cm^−1^ for all samples, respectively (Figure S3, Supporting Information).^[^ ^22^ ^]^ The higher intensity ratios of the D and G bands (*I*_D_/*I*_G_) on PGAs (≈0.88–0.94) than that of GA (0.71) indicated more defects induced by P doping.^[^ ^29^ ^]^ The broad peak centered at 2800 cm^−1^ was attributed to the 2D band, suggesting the development of graphene structure.^[^ ^30^ ^]^ The N_2_ adsorption/desorption isotherms of PGAs were typical IV‐type (Figure S4, Supporting Information), the rapid increase of N_2_ uptake at low‐pressure ranges (P/P_0_ < 0.01) implied the existence of abundant micropores, whereas the hysteresis desorption loop indicated the existence of mesopores.^[^ ^31^, ^32^ ^]^ The highest Brunauer–Emmett–Teller (BET) specific surface area of 548 m^2^ g^−1^ was obtained on PGA‐2, due to the proper pore creation effect of phosphoric acid. Nevertheless, due to the excessive doping of phosphoric acid in PGA‐3, the collapse of porous structure occurred and the specific surface area decreased to 481 m^2^ g^−1^. In contrast, GA showed the lowest BET surface area of 138 m^2^ g^−1^ and the absence of mesopores. The hierarchical pore systems together with high surface areas on PGAs are beneficial for mass transfer and electrolyte diffusion during the CO_2_RR (Figure S5, Supporting Information). The X‐ray photoelectron spectroscopy (XPS) survey confirmed the coexistence of C, O, and P (Figure S6, Supporting Information), the P content increased from 1.78 at% (PGA‐1) and 2.31 at% (PGA‐2) to 2.71 at% on PGA‐3. The high resolution of P 2p spectra can be divided into two peaks located at 132.2 and 133.8 eV, corresponding to the P─C and P─O bond, respectively (Figure 1f).^[^ ^16^ ^]^ Deconvoluted high‐resolution C 1s spectra displayed the C─P bond at 283.8 eV on PGAs, but were invisible for GA (Figure S7, Supporting Information).^[^ ^29^ ^]^ As for the high‐resolution O 1s spectra, three bands could be resolved at 533.3, 531.7, and 529.9 eV, representing hydroxyl groups, C─O, and C─O bonds, respectively (Figure S8, Supporting Information).^[^ ^20^ ^]^

### 2.2 Electrochemical Characterization

**Figure 2:**
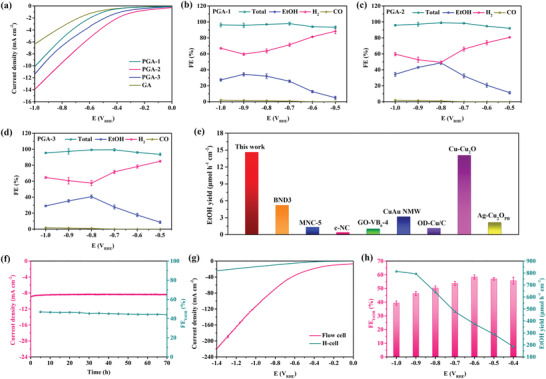
a) LSV curves tested in CO_2_‐saturated 0.5 M KHCO_3_ solution for all samples. FEs of all products at different potentials on b) PGA‐1, c) PGA‐2, and d) PGA‐3. e) Comparison of EtOH yield with different catalysts. f) Stability test on PGA‐2. g) LSV curves of PGA‐2 tested in H‐cell and flow cell. h) EtOH FE and yield on PGA‐2 in flow cell.

The CO_2_RR electroactivity was evaluated in an H‐type three‐electrode cell with a CO_2_‐saturated 0.5 m KHCO_3_ solution (Figure S9, Supporting Information). It should be noted that the PGAs were compressed to working electrodes instead of coating electrode inks. All potentials were versus reversible hydrogen electrode (RHE) hereafter. The linear sweep voltammetry (LSV) showed larger current densities in CO_2_‐saturated electrolyte than those in Ar‐saturated electrolyte on all samples (Figure S10, Supporting Information), signifying the favorable occurrence of CO_2_RR besides HER. Notably, the low onset potentials and high current densities for CO_2_ reduction on PGAs, compared to those of GA, suggested the crucial role of P dopant in promoting CO_2_RR (**Figure** **2**a). The liquid and gaseous products were detected by ^1^H nuclear magnetic resonance (NMR) spectroscopy and on‐line gas chromatography (GC), respectively. As shown in Figure 2b–d, EtOH as the only liquid product and a trace amount of CO were generated on PGAs with total FEs close to 100%. In particular, PGA‐2 delivered the highest EtOH FE of 48.7% at −0.8 V (Figure 2c) with a EtOH partial current density (*j*_EtOH_) of 4.7 mA cm^−2^ (Figure S11, Supporting Information). Notably, such high *j*_EtOH_ and EtOH FE set the new benchmark for electrochemical CO_2_‐to‐EtOH conversion (Table S2 and Figure S12, Supporting Information). In contrast, PGA‐1 and PGA‐3 displayed a maximum EtOH FE of 34.3% at −0.9 V (Figure 2b) and 40.7% at −0.8 V (Figure 2d), respectively. It is worth noting that, within the whole applied potential range, the CO generation was greatly suppressed with Fes less than 1.9%. Meanwhile, EtOH could not be detected on GA, while CO was the only product from CO_2_RR with Fes ranging from 15.6% to 30.1% (Figure S13, Supporting Information). The cathodic energy efficiency (EE) was calculated to evaluate the energy conversion efficiency for EtOH production. As shown in Figure S14 (Supporting Information), the maximum EE of 27.3% was achieved at −0.8 V on PGA‐2, which was comparable to that of reported state‐of‐the‐art catalysts (Table S2, Supporting Information). Furthermore, PGA‐2 delivered the highest EtOH yield of 15.7 µmol h^−1^ cm^−2^ at −0.9 V, which is 1.9‐ and 1.6‐times higher than that of PGA‐1 (8.1 µmol h^−1^ cm^−2^) and PGA‐3 (9.6 µmol h^−1^ cm^−2^), respectively (Figure S15, Supporting Information). To the best of our knowledge, the EtOH yield of 15.7 µmol h^−1^ cm^−2^ on PGA‐2 is the highest compared to previously reported EtOH‐selective electrocatalysts (Figure 2e; and Table S2, Supporting Information).

Besides the high electrochemical activity, long‐term stability was considered and evaluated. As shown in Figure 2f, no deterioration in both current density and EtOH FE can be observed during the prolonged electrolysis on PGA‐2 at −0.8 V for 70 h. Moreover, negligible changes in XRD patterns, SEM/TEM images, and XPS analysis after the durability test demonstrated the excellent structural stability of PGA‐2 (Figure S16, Supporting Information). To confirm the origin of EtOH during CO_2_RR, sufficient control experiments were carried out. When the feed gas was switched to Ar, no reduction product can be detected (Figure S17, Supporting Information). Meanwhile, the isotope‐labeling experiment was performed using ^13^CO_2_ feedstock to ascertain the actual carbon source. From the ^1^H NMR spectra, the signals of ethanol split into two peaks by the coupling with ^13^C atoms (Figure S18a, Supporting Information).^[^ ^3^ ^]^ Moreover, the ^13^C NMR spectra showed strong ^13^C signals as well (Figure S18b, Supporting Information). These results suggested that EtOH was obtained from the reduction of CO_2_ rather than other carbonaceous sources.^[^ ^1^, ^25^ ^]^

To understand the origin of high catalytic activity on PGAs, electrochemical surface areas (ECSA) were estimated from the double‐layer capacitance (*C*_dl_) by using cyclic voltammograms (Figure S19, Supporting Information).^[^ ^6^, ^9^ ^]^ As expected, PGA‐2 possessed the largest *C*_dl_ (28 mF cm^−2^), higher than that of PGA‐1 (14 mF cm^−2^), PGA‐3 (19 mF cm^−2^), and GA (5 mF cm^−2^), implying abundant accessible surface areas and active sites (Figure S20, Supporting Information). We found that the ESCA values were linearly correlated to the BET specific surface areas (Figure S21, Supporting Information), ascribing to that larger BET specific surface areas can provide more accessible active sites for CO_2_ adsorption and activation.^[^ ^33^, ^34^ ^]^ Moreover, the high CO_2_ uptake of 2.38 mmol g^−1^ on PGA‐2, 3‐folds higher than that of GA (0.81 mmol g^−1^), could provide a high‐concentration CO_2_ environment near the catalyst surface and compensate for the limited CO_2_ solubility in the electrolyte (Figure S22, Supporting Information).^[^ ^26^, ^31^ ^]^ The kinetic insight was illustrated by the Tafel plot, PGA‐2 exhibited the lowest Tafel slope (114 mV dec^−1^) than that of PGA‐1 (150 mV dec^−1^) and PGA‐3 (138 mV dec^−1^), manifesting the accelerated kinetics for CO_2_ reduction to EtOH (Figure S23, Supporting Information). Electrochemical impedance spectroscopy (EIS) exhibited the smaller semicircle radius on PGA‐2, revealing the faster charge transfer at the catalysts/electrolyte interface (Figure S24, Supporting Information).^[^ ^26^ ^]^

To achieve high current densities and overcome limited CO_2_ solubility in aqueous electrolytes, the CO_2_RR performances were further evaluated in a flow cell. PGA‐2 was ground and assembled into a gas diffusion electrode (GDE), and the CO_2_ gas and 1 m KOH electrolyte were continuously circulated in the flow cell. As shown in Figure 2g, the current density could be remarkably improved to 119.5 mA cm^−2^ at −1.0 V and reached 221.6 mA cm^−2^ at −1.4 V. Meanwhile, the EtOH FEs maintained above 40% in the whole applied potential range, the maximum value reached 58.3% at −0.6 V (Figure 2h). Certain amounts of CO, CH_4_, and formate were detected as by‐products (Figure S25, Supporting Information). Moreover, a high EtOH yield of 814 µmol h^−1^ cm^−2^ could be obtained at −1.0 V (Figure 2g). These results indicated the commercial viability of PGA‐2.

### 2.3 In Situ Raman Spectroscopy

**Figure 3:**
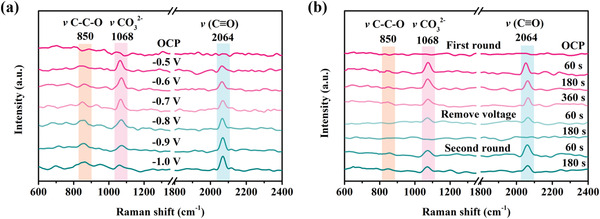
a) Potential‐dependent and b) time‐dependent in situ Raman spectra in CO_2_‐saturated 0.5 m KHCO_3_ solution on PGA‐2.

To elucidate the possible reaction mechanism of CO_2_‐to‐EtOH conversion, in situ Raman spectra were applied on PGA‐2 to detect the reaction intermediates. Potential‐dependent spectroscopy results covering the potential window from the open circuit potential (OCP) to −1.0 V showed signals at 1068 cm^−1^, which can be assigned to the stretching mode of the adsorbed carbonate (CO_3_^2−^ symmetric stretching mode, *v* CO_3_^2−^) in the interfacial region (**Figure** **3**a).^[^ ^35^, ^36^ ^]^ Meanwhile, *CO related peaks (C≡O stretching) at 2064 cm^−1^, essential intermediate to C─C coupling, were also observed.^[^ ^1^, ^37^ ^]^ The signals located at 850 cm^−1^ represented the C─C─O symmetric stretching, indicating the successful C─C coupling and EtOH formation.^[^ ^2^ ^]^ Furthermore, time‐dependent in situ Raman exhibited the decreased intensity of *CO and *v* CO_3_^2−^ signal during 0–360 s in the first round, whereas the signal for C─C─O gradually increased (Figure 3b). After removing the voltage for 180 s, the signals from carbonate, *CO, and C─C─O stretching disappeared. Nevertheless, when the voltage was applied again (second round), the above‐mentioned peaks can be restored in a similar manner. These findings confirmed that the surface adsorbed *CO is the key intermediate in CO_2_‐to‐EtOH conversion.

### 2.4 Theoretical Simulations

**Figure 4:**
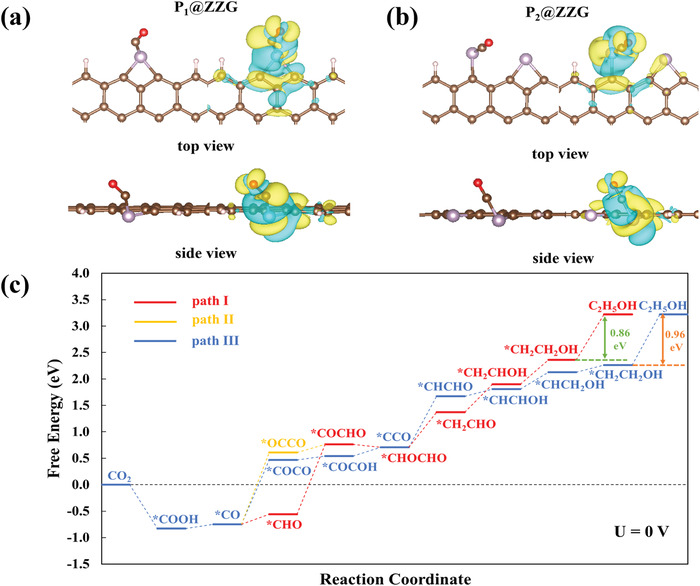
The top and side view of local charge density difference between *CO and basic slabs a) P_1_@ZZG and b) P_2_@ZZG (the left figure is without the iso‐surface and the right is with iso‐surface. Yellow and teal represent the accumulation and depletion of electrons. Isovalue = 0.001; color code: P, purple; H, white; O, red; C, brown). c) Free energy diagram of three possible reaction pathways for CO_2_ reduction to EtOH on P_2_@ZZG at U = 0 V.

Density functional theory (DFT) calculations were carried out to probe the impact of P doping on the preferential production of EtOH. Six possible P‐doped graphene models were constructed (Figure S26, Supporting Information), the active sites and the binding energies of *COOH and *CO intermediates on these models were examined (Figures S27 and S28, Supporting Information). It was found that only P connected with two carbon atoms at the graphene zigzag edge (P_1_@ZZG, Figure S26f, Supporting Information) could chemically bind *CO with negative binding energy, which is necessary for C─C coupling and further conversion to EtOH (**Figure** **4**a; and Figure S28, Supporting Information). In P_1_@ZZG configuration, unsaturated P atoms formed two bonds with neighboring C atoms enabling the stronger *CO adsorption ability than other saturated P‐doped positions (Figure S26a–e, Supporting Information). The felicitous adsorption of *CO could enhance the *CO coverage on P_1_@ZZG surface and promote C─C coupling. In addition, the porous PGAs exhibited abundant defects and boundaries, which could provide more positions to stabilize P atoms in P_1_@ZZG. Although XPS results showed the existence of P─O bonds, the *CO intermediates cannot be adsorbed onto these structures (Figure S28, Supporting Information).

Nevertheless, the *COOH can be strongly adsorbed on P_1_@ZZG with a binding energy of −2.56 eV (Figure S27f, Supporting Information), an endothermic process with a high energy barrier of 1.25 eV was estimated for the conversion of *COOH to *CO, indicating the thermodynamically unfavorable CO_2_RR process (Figure S29, Supporting Information). Therefore, the modified structure was constructed by adding a second P atom in P_1_@ZZG to provide more chemisorption sites for CO_2_ and facilitate the formation of C─C coupling.^[^ ^36^, ^38^ ^]^ After structural optimization, the second P atom only showed a single bond connected to the edge of graphene (Figure S30, Supporting Information). For the bond formed between the P atom (the active site) and C atom of *CO, the length was measured to be 1.814 Å for P_1_@ZZG and 1.680 Å for P_2_@ZZG, indicating the stronger adsorption of *CO on P_2_@ZZG (Figure 4a,b). The more positive charge on the single‐bonded P atom (+0.84) than the double‐bonded P atom (+0.62) demonstrated the higher electron‐withdrawn ability from *CO (Figure S31, Supporting Information). In addition, on P_2_@ZZG, the electron donation was from the C atom of *CO to the O atom of *CO and P atom (Figure 4b), in contrast, the electrons were donated from P atom to C and O atoms of *CO in P_1_@ZZG (Figure 4a). Consequently, the binding energies of *COOH and *CO were calculated to be −3.91 and −2.67 eV on P_2_@ZZG, much lower than those of P_1_@ZZG (−2.56 and −0.03 eV), affording a high possibility for C─C coupling.

To determine the optimized reaction pathway, three proton–electron transfer processes for EtOH production were assessed. The free energy diagrams illustrated that the shared rate‐determining step (RDS) was the final protonation step (Figure 4c), in which EtOH is desorbed with an energy barrier of 0.86 eV for path I and path II and 0.96 eV for path III. Since path I and path II showed the same energy barrier for the RDS, the C─C coupling step was further considered as the second RDS, whose energy barrier was 0.68 eV for path II and 0.66 eV for path I (Figure S32, Supporting Information). Therefore, path I with the C─C couple route of *CO → *CHO → *COCHO was selected as the most thermodynamically preferred route. The favorable path I, highlighted in red, went through in the sequence of CO_2_ → *COOH → *CO → *CHO → *COCHO → *CHOCHO → *CH_2_CHO → *CH_2_CHOH → *CH_2_CH_2_OH → CH_3_CH_2_OH (physical adsorption, Figure 4c; and Figure S33, Supporting Information). In addition, all elementary steps from CO_2_ to EtOH were thermodynamically downhill with an applied voltage of −0.86 V_RHE_ (Figure S34, Supporting Information), which is close to the experimental results. The main competing C_2_ product of C_2_H_4_ shares most reaction intermediates until *CH_2_CHO (or *CH_2_CHOH).^[^ ^2^, ^37^ ^]^ The energy barriers for C_2_H_4_ generation were further calculated to get insight into the suppressed C_2_H_4_ production. As shown in Figure S35 (Supporting Information), *CH_2_CHO spontaneous transformed to *CH_2_CHOH for EtOH generation, while the energy barrier for *CH_2_CHOH→ *CH_2_CH (1.37 eV) is much higher than that of *CH_2_CHOH→ *CH_2_CH_2_OH (0.45 eV). These results confirmed the inhibited C_2_H_4_ generation and the high selectivity for EtOH. Besides, the competing HER was also evaluated, the higher energy barrier than that of CO_2_RR suggested the suppressed HER activity (Figure S36, Supporting Information).

## 3 Conclusion

In summary, P‐doped graphene aerogel was successfully prepared as a self‐supporting electrocatalyst for electroreducing CO_2_ to ethanol. Notably, at −0.8 V_RHE_, the EtOH FE reached 48.7% and maintained excellent stability for 70 h. Meanwhile, a benchmark EtOH yield of 14.62 µmol h^−1^ cm^−2^ was obtained. In situ Raman spectra suggested that the adsorbed *CO intermediates were crucial for ethanol formation. Moreover, DFT calculations demonstrated that the graphene zigzag edge configuration with P active sites could enhance the binding energy for *CO and increase the coverage of *CO on the catalyst surface, thus facilitating the C─C coupling to form *COCHO and following proton–electron transfer processes to generate EtOH. Besides, the well‐developed hierarchically porous structures synergistically rendered abundant accessible active sites and accelerated mass transfer for CO_2_ reduction. This work inspires designing metal‐free based catalysts for the conversion of CO_2_ into high‐valued C_2_ products.

## 4 Experimental Section

The Experimental Section is available in the Supporting Information.

### Statistical Analysis

Statistical analyses were performed using the Origin (Version 9.0). Values for faradaic efficiencies are presented as the mean ± standard deviation. DFT calculations were executed using VASP (Version 5.4.4).

## Conflict of Interest

The authors declare no conflict of interest.

## Supporting information

Supporting Information

## Data Availability

The data that support the findings of this study are available from the corresponding author upon reasonable request.
