# Supplementary material for: Phosphorus‐Doped Graphene Aerogel as Self‐Supported Electrocatalyst for CO2‐to‐Ethanol Conversion
Source: Adv Sci (Weinh). 2022 Jul 12;9(25):2202006. doi: 10.1002/advs.202202006 (PMC9443446; doi:10.1002/advs.202202006)
Supplement: Supplementary file 1 — Supporting Information [file ADVS-9-2202006-s001.pdf]

Supporting Information

Phosphorus-Doped Graphene Aerogel as Self-supported Electrocatalyst for CO_2-to-Ethanol Conversion

Fangqi Yang, Caihong Liang, Haoming Yu, Zheling Zeng, Yeng Ming Lam,* Shuguang Deng, and Jun Wang*

Table of Contents

S1. Experimental and DFT calculation details. 3-7

S2. Schematic illustration of the synthesis process. 8

S3. Textural properties of catalysts. 8-11

S4. Electrochemical tests for all catalysts. 12-15

S5. Characterization for PGA-2 after CO_2 reduction. 15

S6. NMR characterization after electrolysis. 16

S7. Electrochemical behaviors analysis for all catalysts. 17-19

S8. Flow cell tests for PGA-2. 20

S9. DFT calculations on P-doped graphene configurations. 21-28

S10. Electrode potentials for CO_2RR half-reactions in aqueous solution. 29

S11. Comparison of catalysts performance for CO_2 reduction to EtOH......................30

References. 31-34

S1. Experimental and DFT calculation details.

Materials. Graphite powder and potassium bicarbonate ($KHCO_3$) were purchased from Aladdin Reagent Co., Ltd. Phosphoric acid (H_3PO_4) was obtained from Sinopharm Chemical Reagent Co., Ltd. All the chemicals were reagent grade and used as received without further purification. Carbon paper (HCP 030) and Nafion solution (5 wt%) were acquired from Shanghai Hesen Electric Co., Ltd and Sigma-Aldrich, respectively. Ultra-high purity carbon dioxide (99.999%) and argon (99.999%) were supplied from Nanchang Guoteng Gas. Co., Ltd. Ultrapure Millipore water (18.2 MΩ) was supplied by a UP water purification system.

Sample preparation. Graphene oxide (GO) was obtained through chemical exfoliation of graphite powders using the modified Hummer's method.[1] P-doped graphene aerogels (PGAs) were synthesized using the hydrothermal method. Typically, a certain amount of phosphoric acid was mixed with 30 mL GO aqueous dispersion (2 mg mL^{-1}). The mixture was sonicated for 1 h to form a uniform suspension, and then transferred into a 50 mL Teflon-lined stainless-steel autoclave and heated at 180 ℃ for 12 h. After cooling to room temperature, the produced hydrogel was washed with water and ethanol, then freeze-dried. Finally, the product was annealed at 900 ℃ for 1 h under N_2 flow. The sample prepared with 1-, 2-, and 3-mL phosphoric acid loading was denoted as PGA-1, PGA-2, and PGA-3, respectively. Further improving phosphoric acid loading will cause the deformation of aerogel. For comparison, the control sample of GA was prepared without phosphoric acid *via* the same procedure.

Electrochemical measurements. The electrochemical performances were determined using a CHI 660E electrochemical working station with a three-electrode H-cell. The cathodic and anodic compartments were separated by the Nafion®117 membrane. An Ag/AgCl electrode and a graphite rod are served as the reference and counter electrode, respectively. The self-supporting PGAs and GA can be cut into the desired size and directly used as the working electrode (Figure S9). All potentials were measured against the Ag/AgCl reference electrode and converted to the reversible hydrogen electrode (RHE) using the equation of $E\ (V_{RHE}) = E\ (V_{Ag/AgCl}) + 0.21\ V + 0.0591 \times pH$. The electrolysis was conducted in a CO_2-saturated 0.5 M $KHCO_3$ solution (pH = 7.2) at ambient temperature and pressure. During electrolysis, CO_2 was continuously bubbled into the cathodic compartment at a rate of 20 sccm. The gas products were measured using on-line gas chromatography (GC, Agilent 7890B). The electrolyte after electrolysis was collected and tested by ^1H nuclear magnetic resonance (NMR, Bruker 600 MHz) using a pre-saturation method to suppress the water peak.

For the flow cell test, an Ag/AgCl electrode and Pt foil were used as the reference and counter electrode, respectively. PGA-2 was ground and loaded on a gas diffusion layer (GDL) as the working electrode and 1.0 M KOH (pH = 14.0) was used as the electrolyte. During the tests, the electrolyte was circulated through the cathode compartment at a rate of 12 mL min^{-1}, and CO_2 gas with a flow rate of 20 sccm was fed to the cathode GDL.

***In-situ* Raman test.** *In-situ* Raman spectroscopy was performed using a Confocal

LabRam HR800 microscope (Horiba Jobin Yvon). Raman signals were collected based on a self-made electrochemical cell, in which graphite rod and Ag/AgCl electrode were used as the counter and reference electrode, respectively. The as-prepared PGA catalyst was used as the working electrode in 0.5 M $KHCO_3$ electrolyte with continuous CO_2 flowing at 20 sccm on the backside. A 50× objective lenses and a laser wavelength of 532 nm were applied.

Characterizations. X-ray diffraction (XRD) patterns were obtained on a Bruker D8 Advance X-ray diffractometer with a Cu target (λ = 1.5418 Å). The spectra of X-ray photoelectron spectroscopy (XPS) were analyzed using a Thermo Fisher Scientific Escalab 250Xi system with a monochromatic Al-Kα source. The morphologies and microstructures were characterized by scanning electron microscopy (SEM, Hitachi SU800) and transmission electron microscopy (TEM, FEI Talos F200X). Raman spectra were collected using a LabRam HR800 spectrometer (Horiba Jobin Yvon) with a 532 nm laser source. N_2 and CO_2 adsorption-desorption isotherms were measured on a Micromeritics ASAP 2460 apparatus. Before each adsorption measurement, the sample was degassed at 150 ℃ for 12 h.

Calculation of cathodic energy efficiency (EE).

$$\mathbf{EE} = \frac{\mathbf{1.23 - E^0}}{\mathbf{1.23 - E}} \times \mathbf{FE_{EtOH}}$$

Where E is the applied potential in the experiment, FE_{EtOH} is the Faradaic efficiency of ethanol, E^0 is 0.09 V_{RHE} for the thermodynamic potential of CO_2 reduction to ethanol.

DFT calculations. DFT calculations were executed by VASP with the GGA-PBE

method (generalized gradient approximation with Perdew, Burke, and Ernzerh) functional.[2-4] The cutoff energy, energy convergence, and force convergence were set as 500 eV, 1×10^{-4} eV, and 0.03 eV/Å, respectively. Meanwhile, the gamma point is utilized in Mohkhorst-Pack (MP) grid.[5] In addition, the DFT-D3 method with Becke-Jonson damping was conducted for all calculations.[6]

For all absorbed intermediates of CO_2RR and HER, the binding energy (BE) can be written as:

$$BE = E_{total} - (E_{slab} + E_{ads})$$

Wherein, Et_{otal} is the whole energy of intermediates absorbed on the slab, E_{slab} is the energy of the basic slabs, and E_{ads} represents the energy of various intermediates.

The variation of Gibbs free energy (ΔG) of each reaction step refers to the calculated hydrogen electrode[7] and the expression can be described as:

$$\Delta G = \Delta E + \Delta ZPE - T\Delta S + \Delta G_{pH} + \Delta G_{U}$$

$$\Delta G_{U} = -neU$$

$$\Delta G_{pH} = k_{B}T \times \ln10 \times pH$$

Here, the energy difference of each reaction is ΔE; ΔZPE is the zero-point energy and ΔS is the entropy difference at T=298.15 K. ΔZPE and ΔS were obtained with displacement as 0.015 Å for all absorbed intermediates. ΔG_{U} is the contribution of the electrode potential to ΔG. For the symbols in ΔG_{U} formula, n represents the number of transferred electrons in each step and U is the applied electrode potential. ΔG_{pH} is the correction of free energy at given pH. In this study, the environment was slightly alkaline with a pH of 7.2. All the temperature is 298.15K and k_{B} is the Boltzmann

constant.

The energy of H_2O, CO_2, and H_2 is calculated by VASP in a vacuum. The correction of energy is obtained from vaspkit.[8] The temperature is chosen as 298.15 K, the pressure is 0.035 atm for H_2O (l) and 1 atm for CO_2 and H_2. The energy of OH^- is derived from $G(OH^-) = G(H_2O) – 1/2\ G(H_2)$ in pH = 0. ΔZPE and TΔS of absorbed intermediates are acquired from vaspkit with a temperature of 298.15 K.[8]

In this work, the reaction mechanism can be described as,

$$* + 2CO_2\,(g) + H_2O + e^- \rightarrow *COOH + CO_2\,(g) + OH^-$$

$$*COOH + CO_2\,(g) + e^- \rightarrow *CO + CO_2\,(g) + OH^-$$

$$*CO + CO_2\,(g) + H_2O + e^- \rightarrow *CHO + CO_2\,(g) + OH^-$$

$$*CHO + CO_2\,(g) + H_2O + 2e^- \rightarrow *COCHO + 2OH^-$$

$$*COCHO + H_2O + e^- \rightarrow *CHOCHO + OH^-$$

$$*CHOCHO + H_2O + e^- \rightarrow *CH_2CHO + OH^-$$

$$*CH_2CHO + H_2O + e^- \rightarrow *CH_2CHOH + OH^-$$

$$*CH_2CHOH + H_2O + e^- \rightarrow *CH_2CH_2OH + OH^-$$

$$*CH_2CH_2OH + H_2O + e^- \rightarrow CH_3CH_2OH + OH^-$$

Besides, the HER reaction mechanism in alkaline condition is as follows:

$$* + H_2O \rightarrow *H + *OH$$

$$*H + *OH + e^- \rightarrow *H + OH^-$$

$$*H + OH^{-} \rightarrow \frac{1}{2} H_2 + OH^{-}$$

S2. Schematic illustration of the synthesis process.

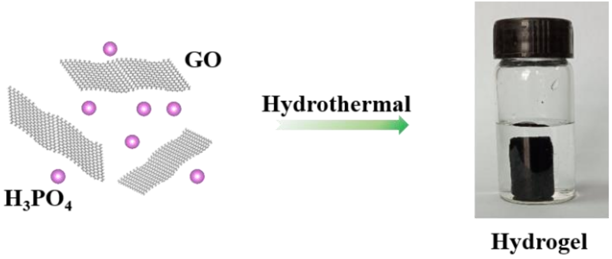

Figure S1. Schematic illustration of P-doped hydrogel preparation.

S3. Textural properties of catalysts.

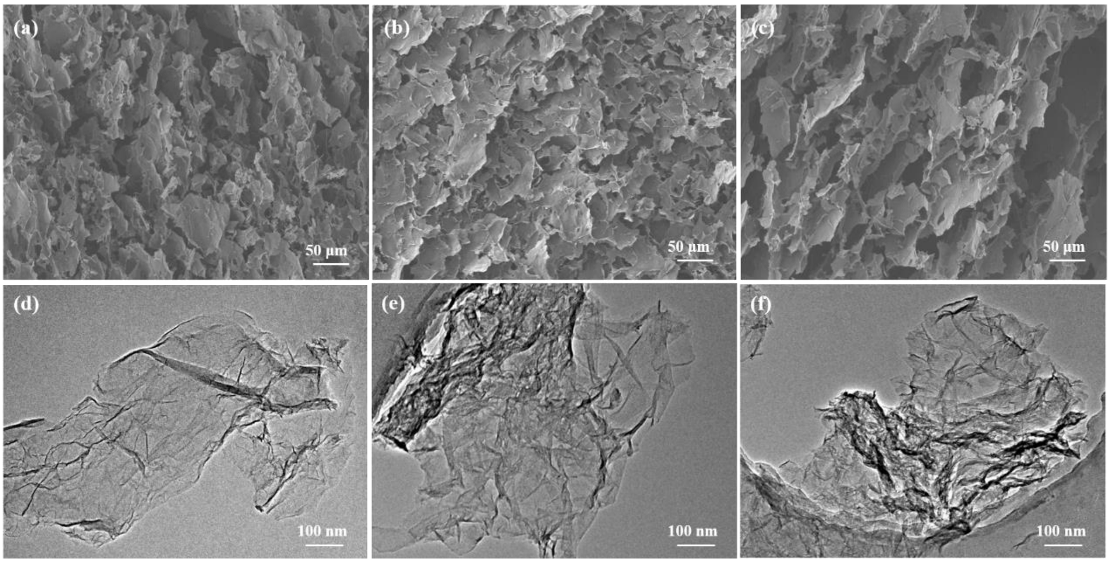

Figure S2. SEM and TEM images of (a and d) GA, (b and e) PGA-1, (c and f) PGA-3.

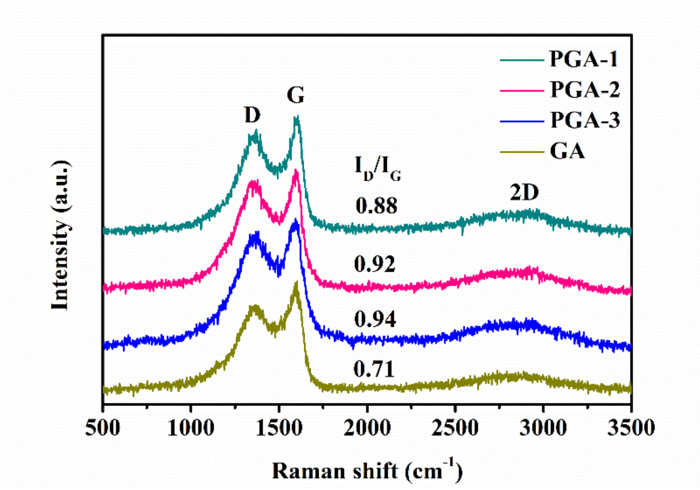

Figure S3. Raman spectra of all samples.

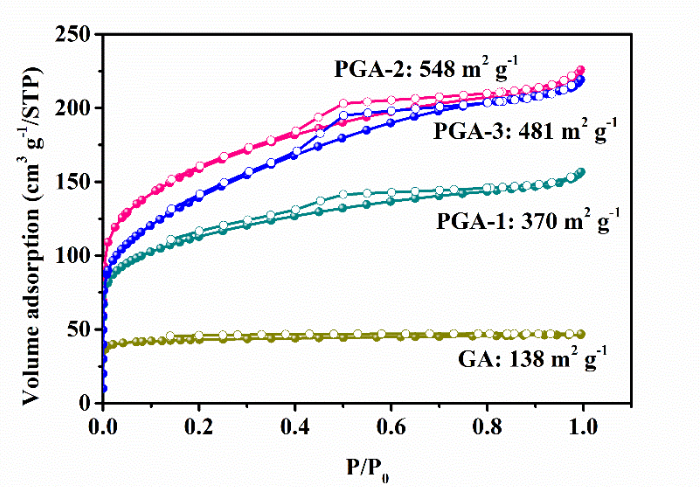

Figure S4. N_2 adsorption-desorption isotherms for all samples.

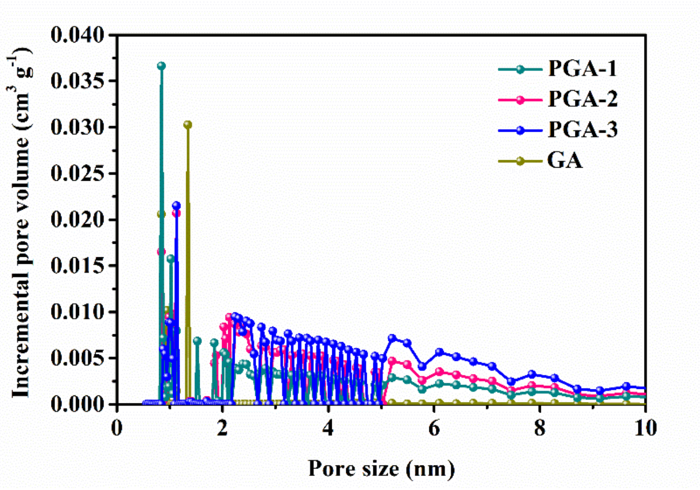

Figure S5. Pore size distribution for all samples.

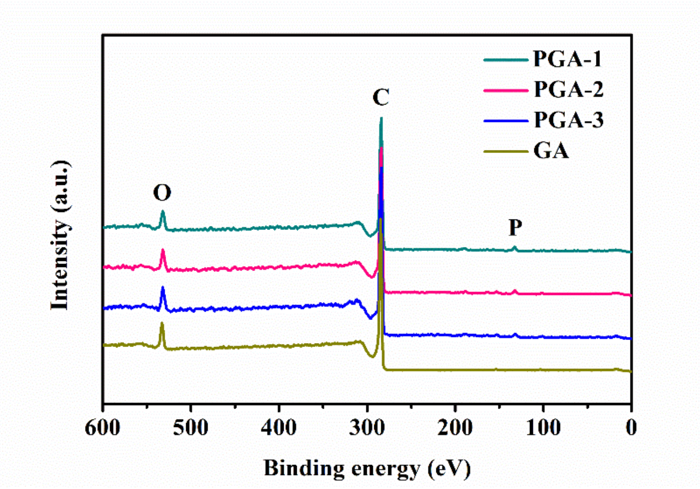

Figure S6. XPS survey for all samples.

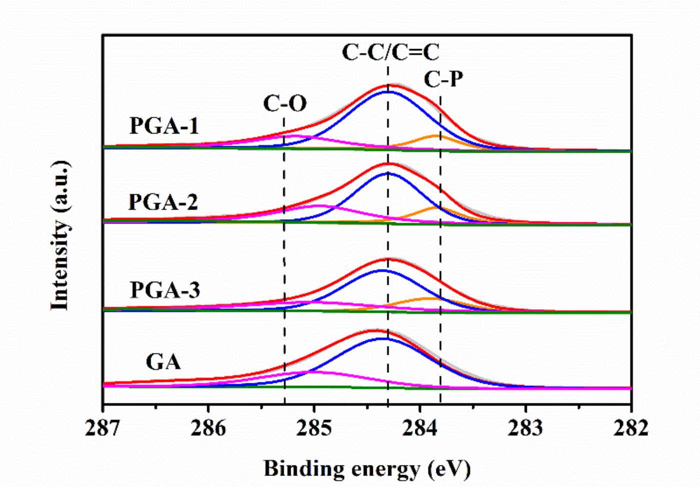

Figure S7. High-resolution C 1s XPS spectra for all samples.

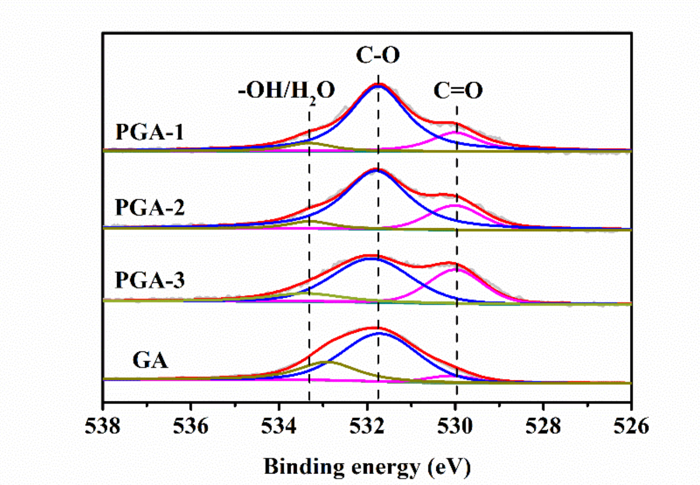

Figure S8. High-resolution O 1s XPS spectra for all samples.

S4. Electrochemical tests for all catalysts.

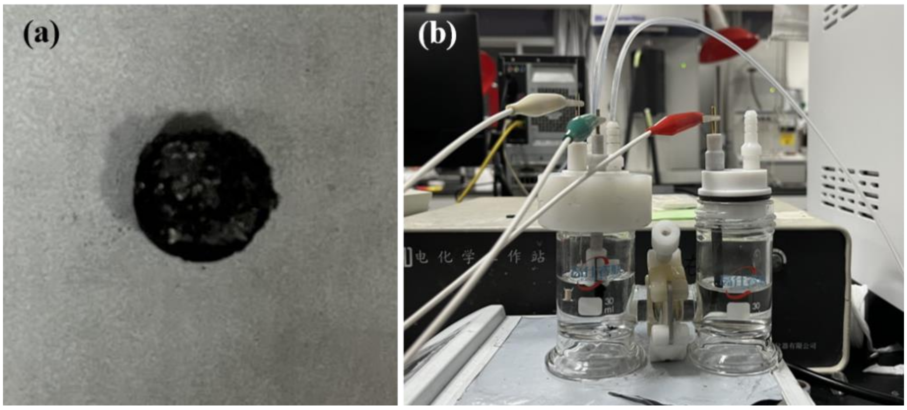

Figure S9. Image of (a) PGA-2 electrode and (b) assembled in H-cell.

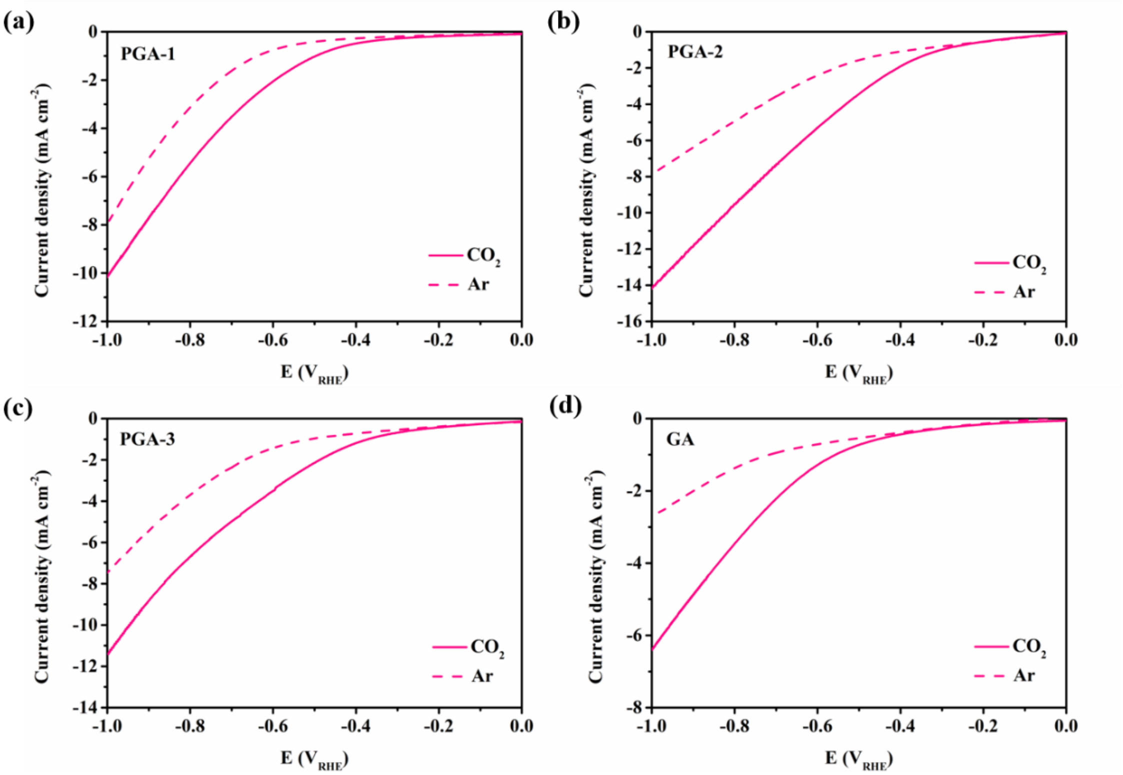

Figure S10. LSV curves in CO_2- and Ar-saturated 0.5 M $KHCO_3$ electrolyte on (a) PGA-1, (b) PGA-2, (c) PGA-3, and (d) GA.

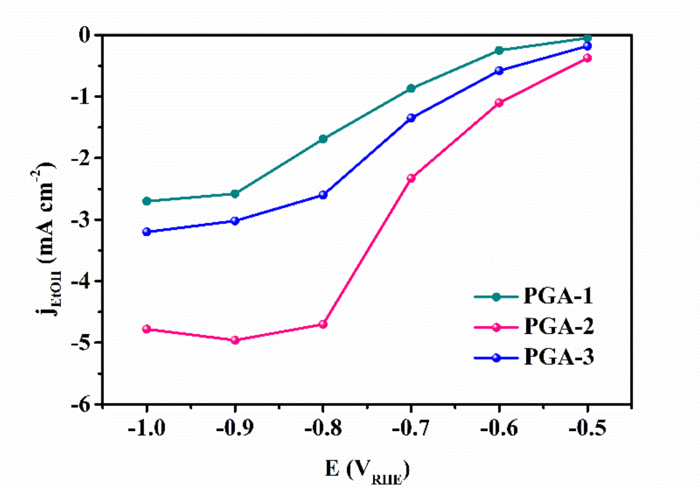

Figure S11. EtOH partial current densities of all samples.

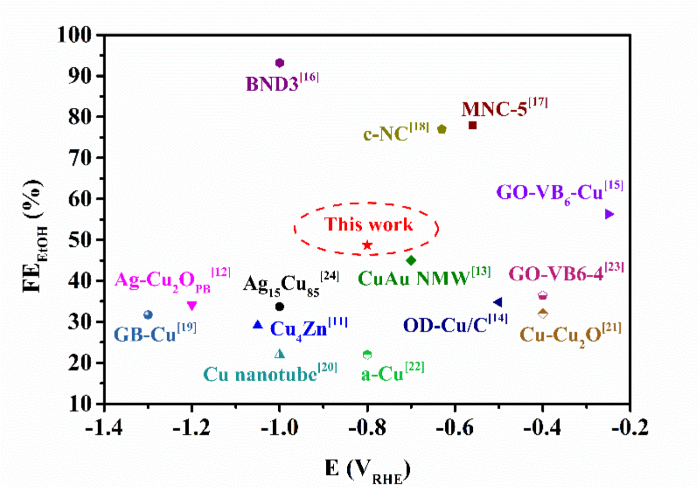

Figure S12. Comparison of EtOH FE on different state-of-the-art catalysts.

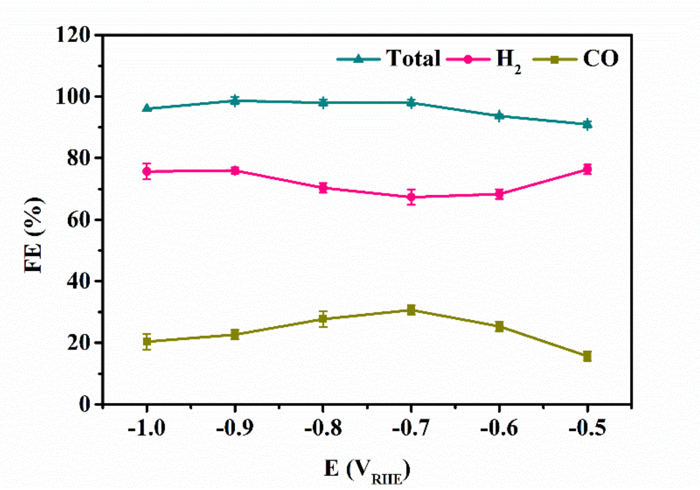

Figure S13. FE of all products at different applied potentials in CO_2-saturated 0.5 M $KHCO_3$ electrolyte on GA.

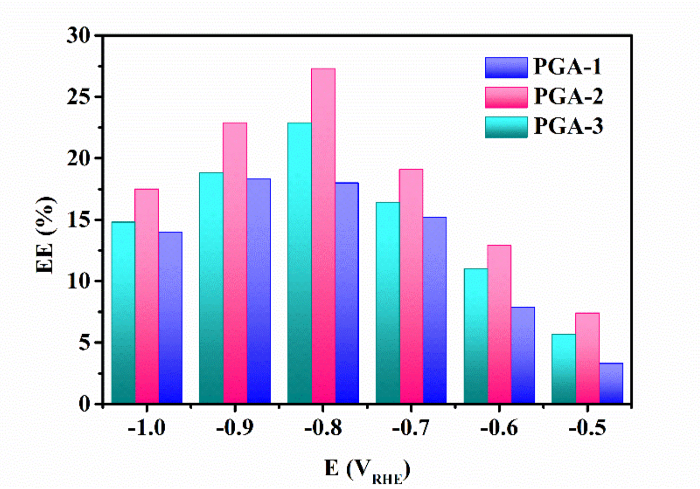

Figure S14. Energy efficiency at different applied potentials for all samples.

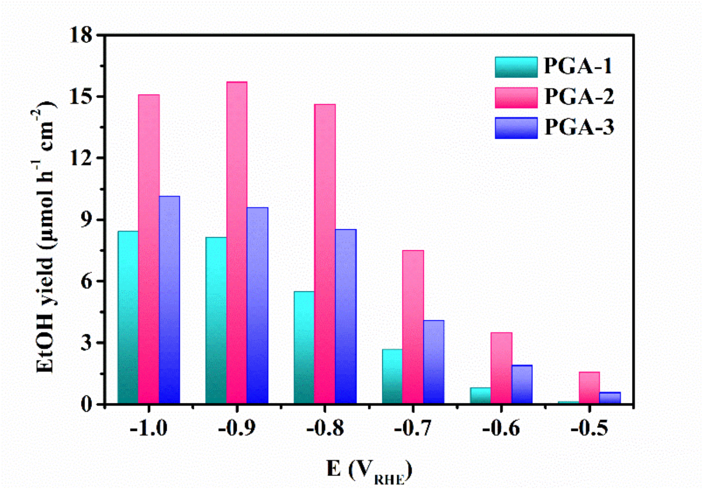

Figure S15. EtOH yields at different applied potentials for all samples.

S5. Characterization for PGA-2 after CO_2 reduction.

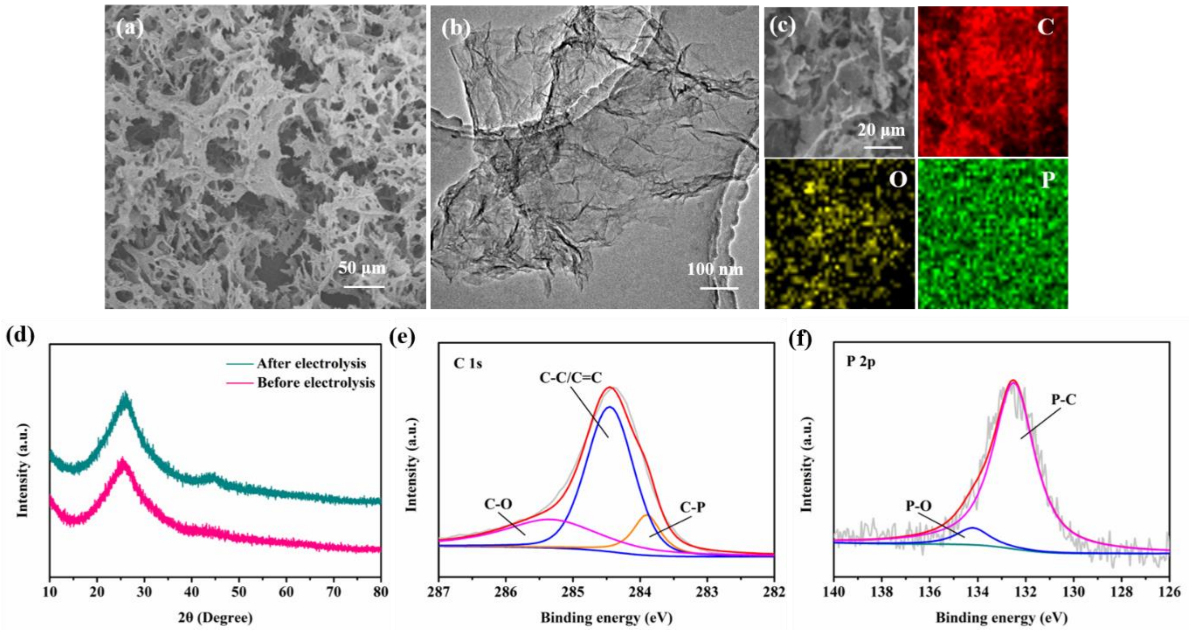

Figure S16. (a) SEM and (b) TEM images, (c) EDS mappings, (d) XRD patterns, and high-resolution XPS spectra of (e) C 1s and (f) P 2p of PGA-2 after duration test.

S6. NMR characterization after electrolysis.

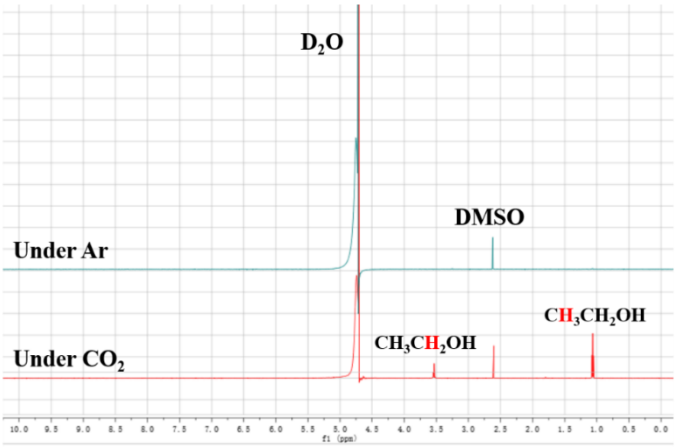

Figure S17. ^{1}H NMR results of the liquid product of PGA-2 in CO_2 and Ar-saturated 0.5 M $KHCO_3$ electrolyte after electrolysis.

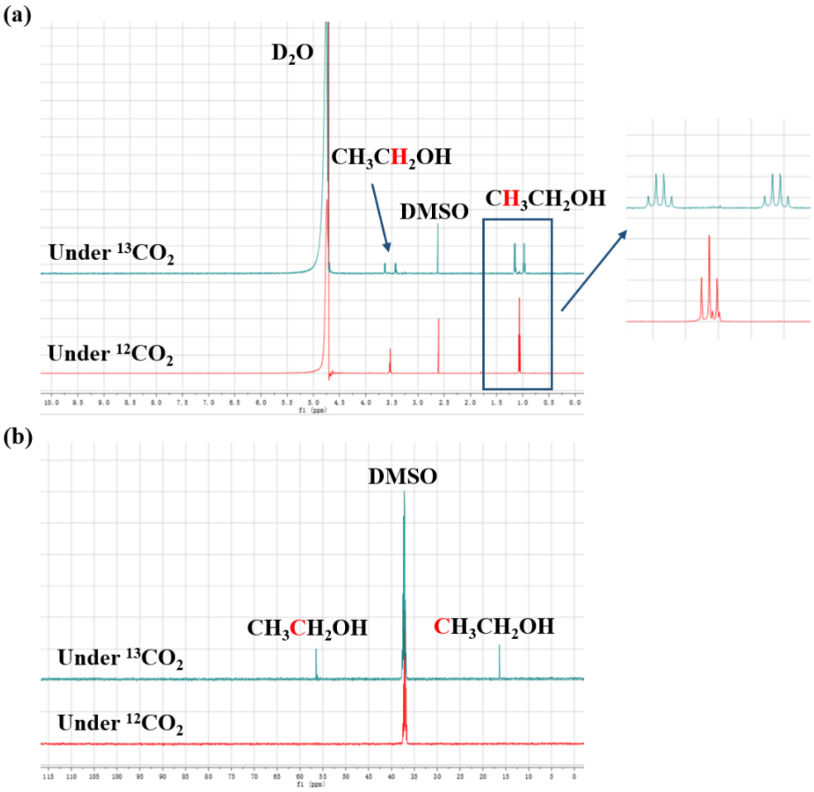

Figure S18. (a) ^{1}H NMR and (b) ^{13}C NMR spectra of the catholyte after electrolysis using $^{13}CO_2$ and $^{12}CO_2$ as feeding gas on PGA-2 at -0.8 V.

S7. Electrochemical behaviors analysis for all catalysts.

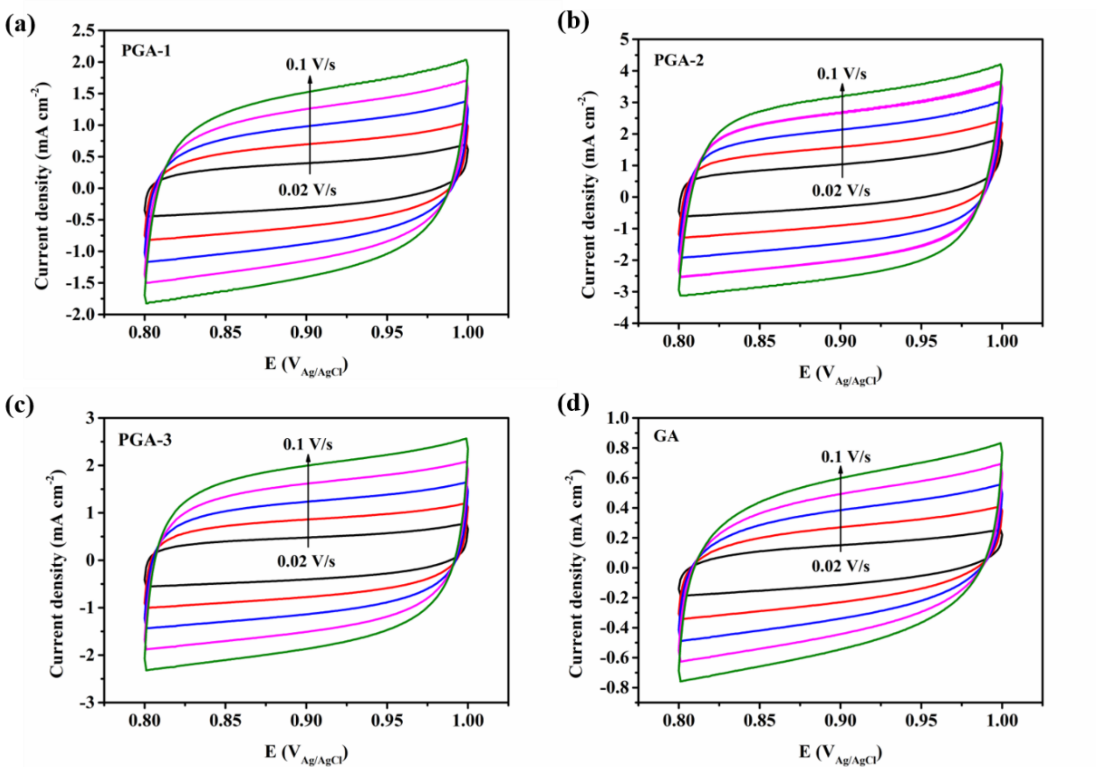

Figure S19. Cyclic voltammetry (CV) results of (a) PGA-1, (b) PGA-2, (c) PGA-3, and (d) GA.

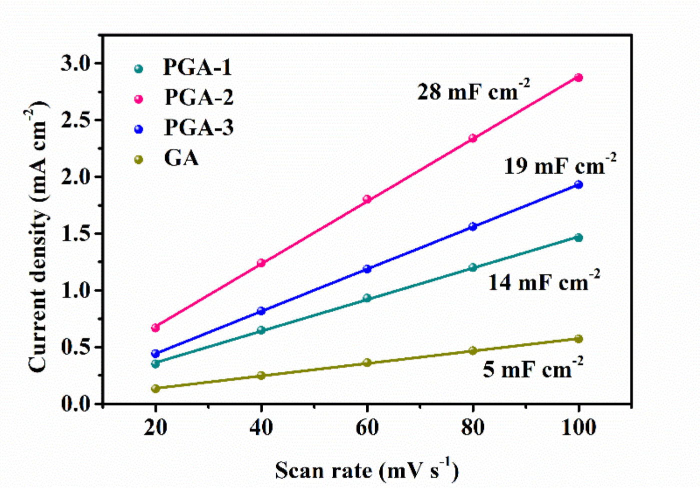

Figure S20. Charging current densities plotted against scan rates for all samples.

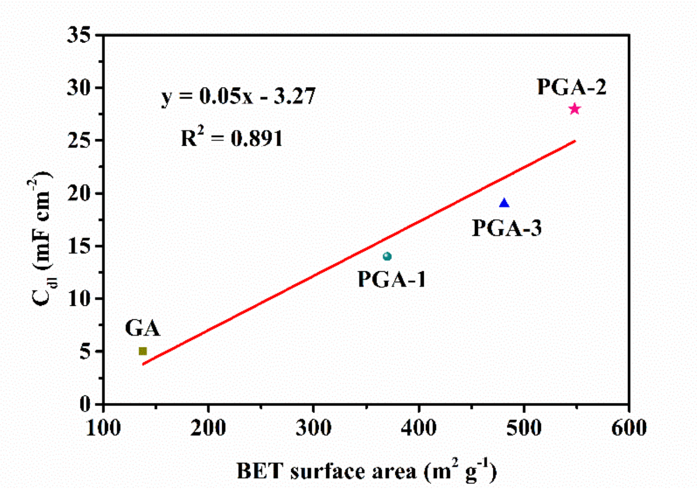

Figure S21. Relationship between C_{dl} and BET specific surface areas.

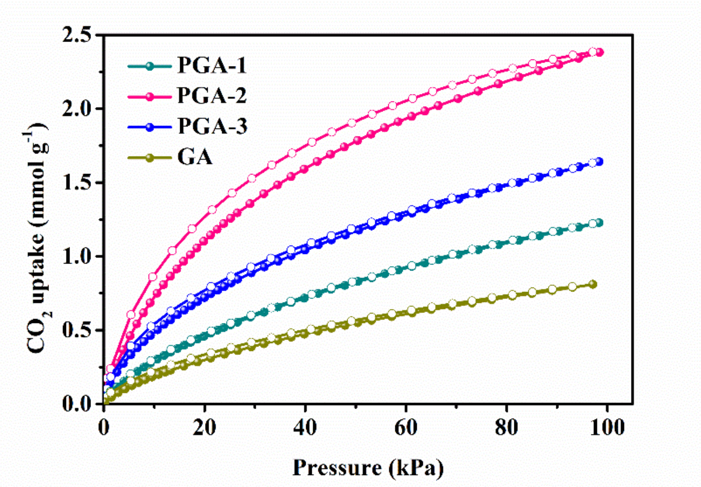

Figure S22. Adsorption (solid) and desorption (open) isotherms of CO_2 at 298 K and 1.0 bar on all samples.

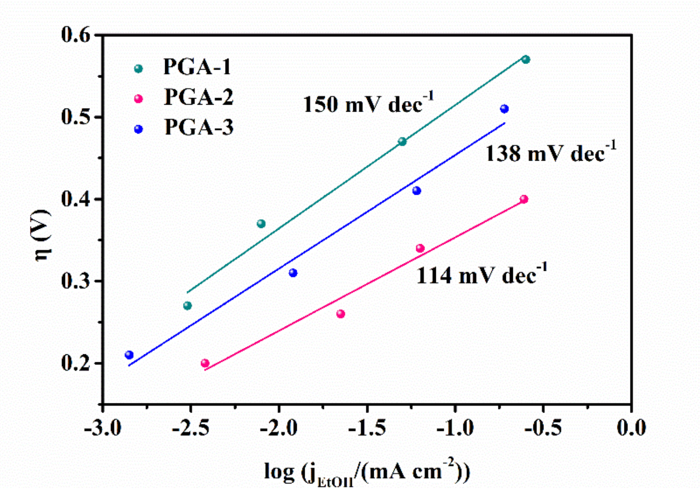

Figure S23. Tafel plot for all samples.

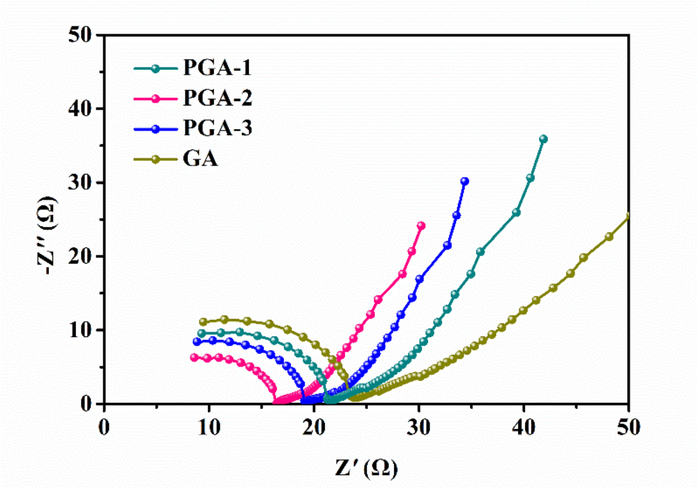

Figure S24. Electrochemical impedance spectroscopy of all samples.

S8. Flow cell tests for PGA-2.

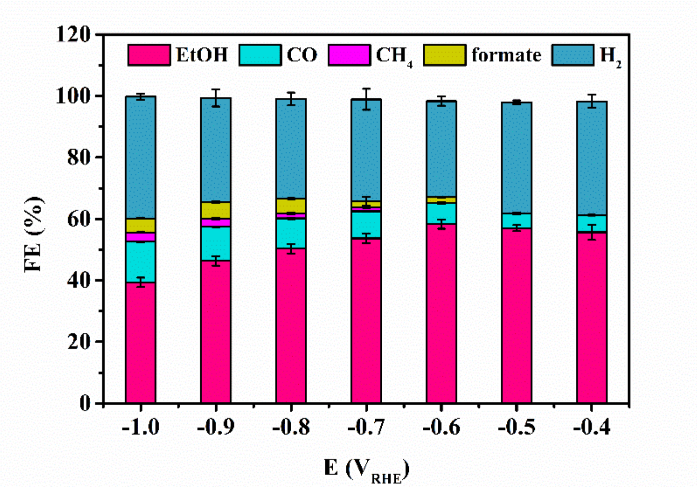

Figure S25. FE of products at different applied potentials on PGA-2 in flow cell.

S9. DFT calculations on P-doped graphene configurations.

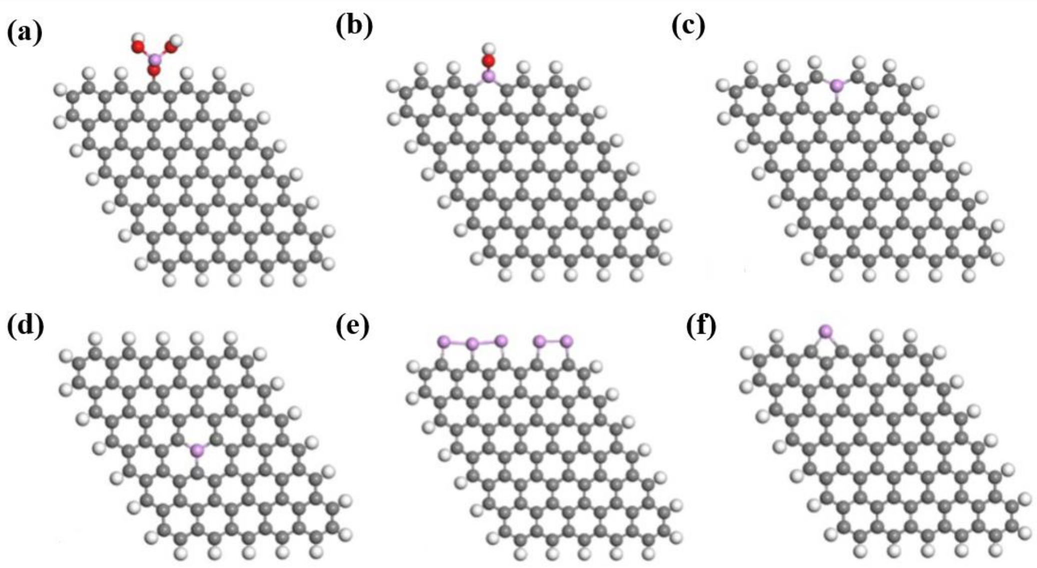

Figure S26. Models of P-doped graphene configurations. (a) $P[OH]_2O$-doped graphene; (b) P[OH]-doped graphene (OH terminated is due to the slightly alkaline environment); (c) P replaces one carbon atom at the boundary; (d) P replaces one carbon atom in the center; (e) P-chain doped graphene; (f) P atom connected at the boundary of graphene (P_1@ZZG). Color code: P, pink; H, white; O, red; C, grey.

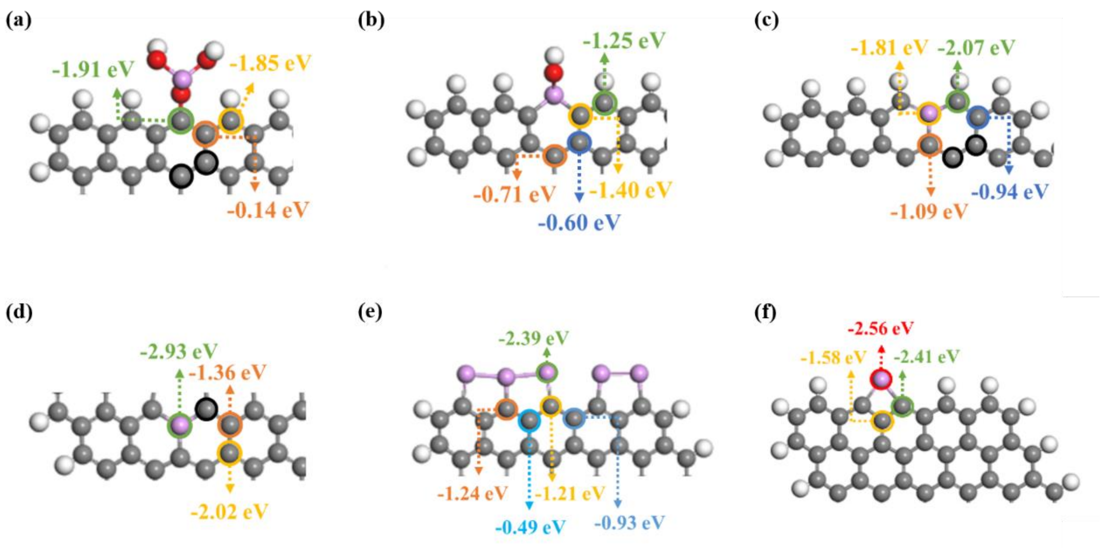

Figure S27. Binding energy of *COOH absorbed at different sites of various configurations. (a) P[OH]$_2$O-doped graphene; (b) P[OH]-doped graphene; (c) P replaces one carbon atom at the boundary of graphene; (d) P replaces one carbon atom in the center of graphene; (e) P-chain doped graphene; (f) P atom connected at the boundary of graphene. Color code: P, pink; H, white; O, red; C, grey.

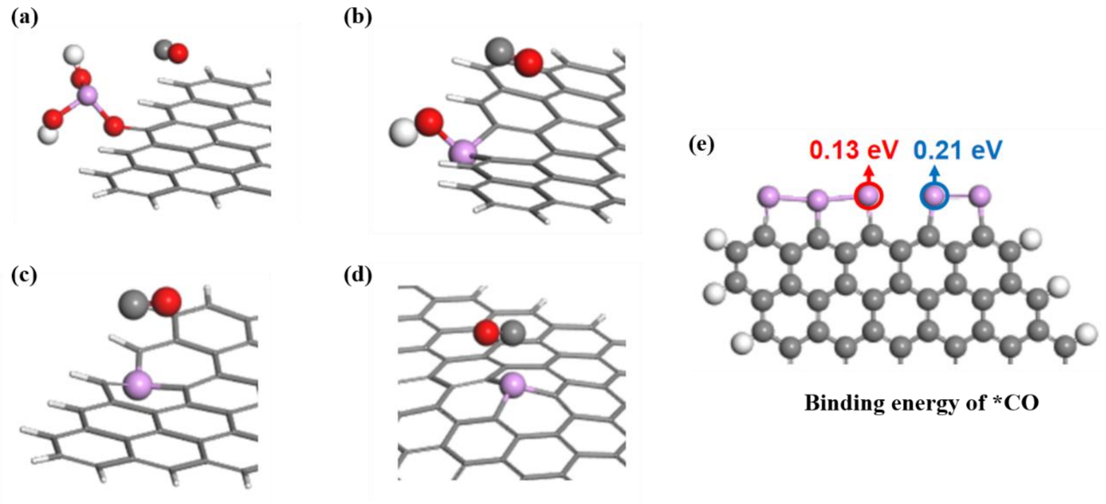

Figure S28. Absorbed situation of *CO at different sites of various configurations. (a) P[OH]$_2$O-doped graphene; (b) P[OH]-doped graphene; (c) P replaces one carbon atom at the boundary of graphene; (d) P replaces one carbon atom in the center of graphene; (e) P-chain doped graphene; Color code: P, pink; H, white; O, red; C, grey.

The absorbed sites on different models for *CO are chosen from the most possible absorbing sites of *COOH. However, *CO cannot be absorbed on models displayed in Figure S26a-d. The binding energies of *CO on the two P sites were all positive as 0.13 and 0.21 eV (Figure S28e). The high *CO binding energies indicated that the process of *COOH conversion to EtOH cannot be conducted. Besides, the *CO absorbed on the slab in Figure S26f was shown in the rightmost picture in Figure S29.

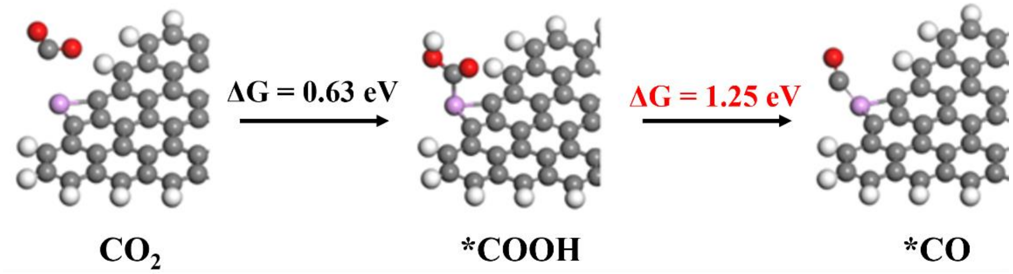

Figure S29. Reaction pathway from CO_2 to *COOH to *CO on P_1@ZZG structure.

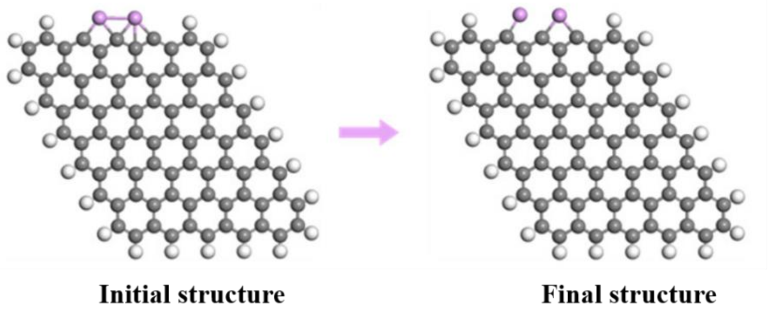

Figure S30. Two P-doped graphene model (P_2@ZZG): the initial constructed structure (left) and the final optimized structure (right).

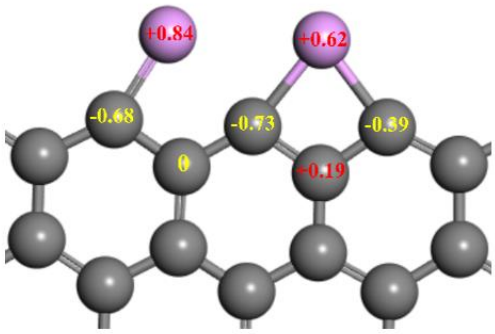

Figure S31. Bader charge analysis around P atoms in P_2@ZZG.

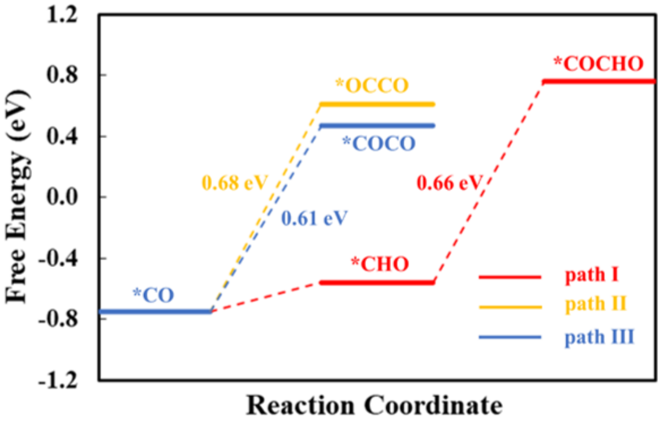

Figure S32. The reaction pathway from *CO to the first C-C coupling intermediates: path I: *CO → *CHO → *COCHO; path II: *CO → *COCO; path III: *CO → *OCCO. Here, the denoted energy barriers are divided by two, because these steps included two transferred electrons.

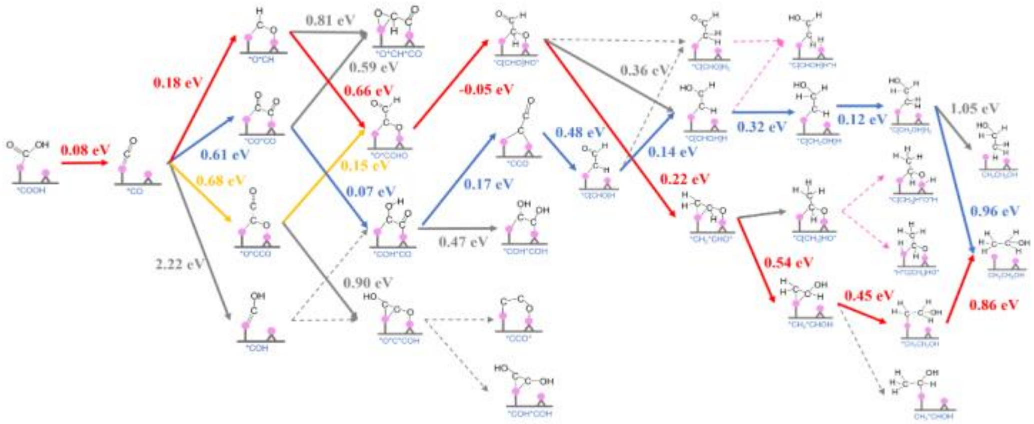

Figure S33. Symbolic element model for the overall pathways (the grey bottom line represents graphene and P atom is the purple circle).

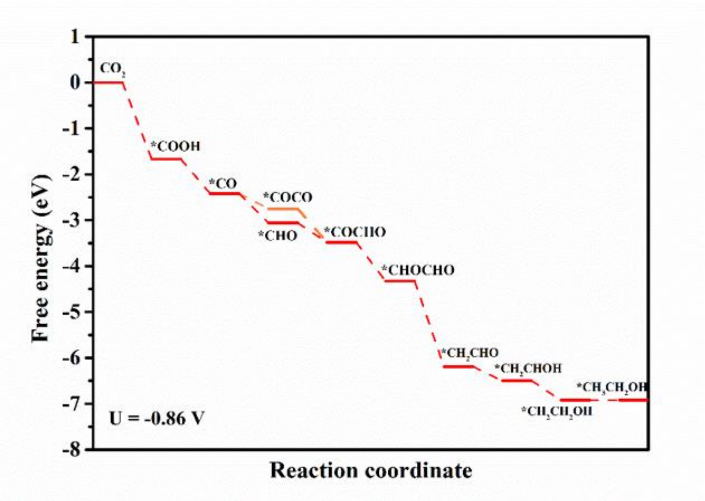

Figure S34. Free energy diagram of two optimal reaction pathways for CO_2 reduction to EtOH on P_2@ZZG at U = -0.86 V.

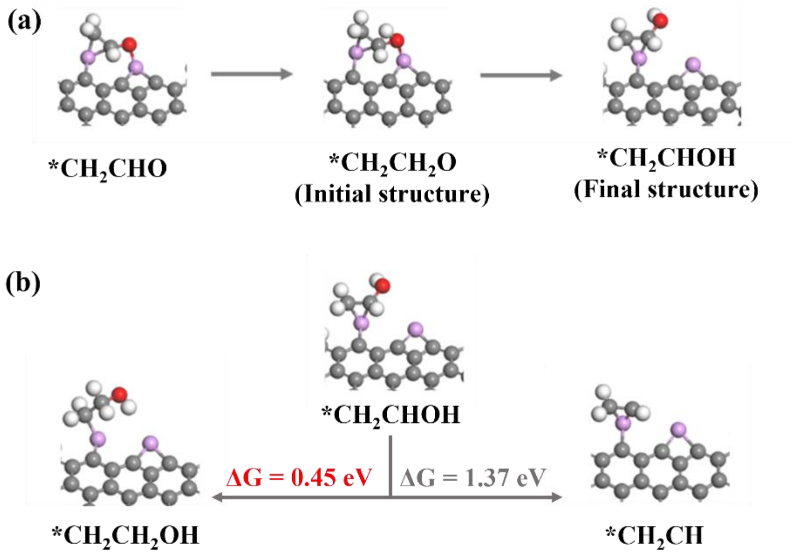

Figure S35. (a) Schematic illustration of $*CH_2CHO$ transformation process on P_2@ZZG. (b) The comparison of energy barrier from $*CH_2CHOH$ to $*CH_2CH_2OH$ or $*CH_2CH$.

C_2H_4 and EtOH share most reaction intermediates until $*CH_2CHO$ (or $*CH_2CHOH$), which could transform to EtOH through further protonation to $*CH_2CHOH$ (or $*CH_2CH_2OH$/$*CH_3CHOH$), or to C_2H_4 *via* $*CH_2CH_2O \rightarrow *O + CH_2CH_2$ (or $*CH_2CHOH \rightarrow *CH_2CH + OH^-$). The $*CH_2CH_2O$ intermediate will spontaneously transform to $*CH_2CHOH$ after optimization, implying the favorable EtOH generation (Figure S35a). For $*CH_2CHOH$, the energy barrier towards $*CH_2CH$ is 1.37 eV, much higher than that of $*CH_2CH_2OH$ (0.45 eV, Figures S33 and 35b). The value is also lower than that of $*CH_3CHOH$ (0.69 eV). Therefore, the whole reaction prefers processing to EtOH rather than C_2H_4.

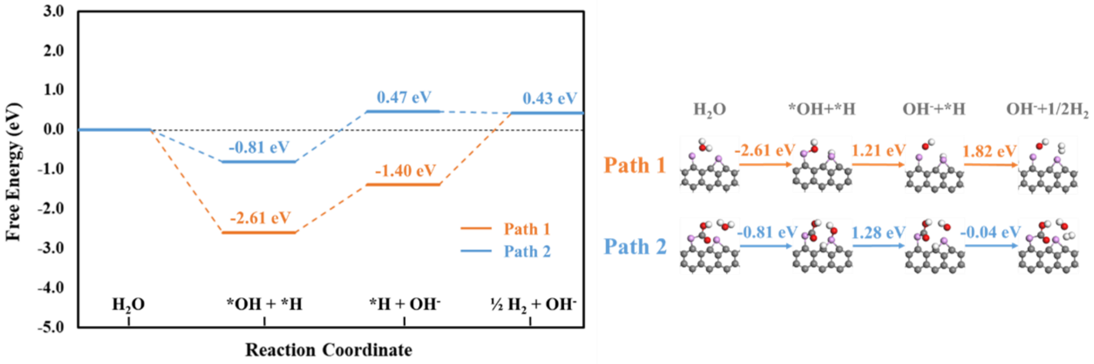

Figure S36. Free energy diagram (left) and corresponding stick-ball models (right) of HER at three different pathways (color code: P, purple; H, white; O, red; C, grey).

In Path 1, water was directly split to *OH and *O on different P atoms with an overall energy barrier of 1.82 eV. In path 2, after adsorbing *COOH onto the single-bond P atom, the overall HER progressed around the neighboring P atom with a lower energy barrier of 1.28 eV that was still higher than that of CO_2RR (0.86 eV). Thus, HER was greatly depressed on P_2@ZZG with the applied voltages around -0.86 V.

S10. Electrode potentials for CO_2RR half-reactions in aqueous solution.

Table S1. Electrode potentials for CO_2RR half-reactions in aqueous solution.[9]

**CO_2RR half-reactions**	**Electrode potentials (V_{RHE}) at pH = 7**
$CO_2 + 2H^+ + 2e^- \rightarrow CO + H_2O$	-0.11
$CO_2 + 2H^+ + 2e^- \rightarrow HCOOH$	-0.22
$CO_2 + 8H^+ + 8e^- \rightarrow CH_4 + 2H_2O$	0.17
$2CO_2 +12H^+ + 12e^- \rightarrow C_2H_4 + 4H_2O$	0.08
$2CO_2 + 12H^+ + 12e^- \rightarrow C_2H_5OH + 3H_2O$	0.08
$3CO_2 + 18H^+ + 18e^- \rightarrow C_3H_7OH + 5H_2O$	0.09

S11. Comparison of catalysts performance for CO_2 reduction to EtOH.

Table S2. Comparison of catalytic performances for CO_2 electroreduction to EtOH.

Catalyst	Electrolyte	E^a (V_{RHE})	FE_{EtOH} (%)	Yield^a (μmol h^{-1} cm^{-2})	$j_{EtOH}{}^a$ (mA cm^{-2})	EE^a (%)
**PGA-2**	**0.5 M $KHCO_3$**	**-0.8**	**48.7**	**14.62**	**4.7**	**27.3**
$Fe_2P_2S_6$ nanosheet [10]	0.5 M $KHCO_3$	-0.2	23.1	1.096	0.01	18.4
Cu_4Zn [11]	0.1 M $KHCO_3$	-1.05	29.1	25.45	8.2	14.6
Ag-Cu_2O_{PB} [12]	0.2 M KCl	-1.2	34.15	2.15	1.02	16.0
CuAu NWA [13]	0.1 M $KHCO_3$	-0.7	45	3.156	0.4	26.6
OD-Cu/C [14]	0.1 M $KHCO_3$	-0.5	34.8	1.12	0.4	21.7
GO-VB_6-Cu [15]	0.1 M $KHCO_3$	-0.25	56.3	8.08	2.55	43.4
BND3 [16]	0.1 M $KHCO_3$	-1.0	93.2	5.2	0.58	47.6
MNC-5 [17]	0.1 M $KHCO_3$	-0.56	78	1.3	0.39	49.7
c-NC [18]	0.1 M $KHCO_3$	-0.63	77	0.38	0.13	47.2
GB-Cu [19]	1 M KOH	-1.3	31.7	44.388	45	14.3
Cu nanocube [20]	0.1 M $KHCO_3$	-1.0	22	21.91	6.89	11.2
Cu-Cu_2O [21]	0.1 M KCl	-0.4	32	11.47	3.68	22.4
a-Cu [22]	0.1 M $KHCO_3$	-0.8	22	0.40	0.91	12.4
GO-VB_6-4 [23]	0.1 M $KHCO_3$	-0.4	36.4	1.014	0.43	25.5
$Ag_{15}Cu_{85}$ [24]	0.5 M $KHCO_3$	-1.0	33.7	26.97	8.67	17.2

[a] The data were obtained at the potential of maximum EtOH FE achieved.

Reference.

[1] D. C. Marcano, D. V. Kosynkin, J. M. Berlin, A. Sinitskii, Z. Sun, A. Slesarev, L. B. Alemany, W. Lu, J. M. Tour. Improved synthesis of graphene oxide. *ACS Nano* **2010**, *4*, 4806-4814.

[2] G. Kresse, J. Hafner. Ab initio molecular dynamics for liquid metals. *Phys. Rev. B* **1993**, *47*, 558-561.

[3] G. Kresse, J. Furthmüller. Efficiency of ab-initio total energy calculations for metals and semiconductors using a plane-wave basis set. *Comput. Mater. Sci.* **1996**, *6*, 15-50.

[4] J.-P. Perdew, K. Burke, M. Ernzerhof. Generalized gradient approximation made simple. *Phys. Rev. Lett.* **1996**, *77*, 3865-3868.

[5] H.-J. Monkhorst, J.-D. Pack. Special points for Brillouin-zone integrations. *Phys. Rev. B* **1976**, *13*, 5188-5192.

[6] S. Grimme. Semiempirical GGA-type density functional constructed with a long-range dispersion correction. *J. Comput. Chem.* **2006**, *27*, 1787-1799.

[7] J.-K. Nørskov, J. Rossmeisl, A. Logadottir, L. Lindqvist, J.-R. Kitchin, T. Bligaard, H. Jónsson, Origin of the overpotential for oxygen reduction at a fuel-cell cathode. *J. Phys. Chem. B* **2004**, *108*, 17886-17892.

[8] V. Wang, N. Xu, J.-C. Liu, G. Tang, W.-T. Geng. A user-friendly interface facilitating high-throughput computing and analysis using VASP code. *Comput. Phys. Commum.* **2021**, *267*, 108033.

[9] A. S. Varela, W. Ju, P. Strasser. Molecular nitrogen-carbon catalysts, solid metal organic framework catalysts, and solid metal/nitrogen-doped carbon (MNC) catalysts for the electrochemical CO_2 reduction. *Adv. Energy Mater.* **2018**, *8*, 1802905.

[10] L. Ji, L. Chang, Y. Zhang, S. Mou, T. Wang, Y. Luo, Z. Wang, X. Sun. Electrocatalytic CO_2 reduction to alcohols with high selectivity over two-dimensional $Fe_2P_2S_6$ nanosheet. *ACS Catal.* **2019**, *9*, 9721-9725.

[11] D. Ren, B. S.-H. Ang, B. S. Yeo. Tuning the selectivity of carbon dioxide electroreduction toward ethanol on oxide-derived CuxZn catalysts. *ACS Catal.* **2016**, *6*, 8239-8247.

[12] S. Lee, G. Park, J. Lee. Importance of Ag-Cu biphasic boundaries for selective electrochemical reduction of CO_2 to ethanol. *ACS Catal.* **2017**, *7*, 8594-8604.

[13] W. Zhu, K. Zhao, S. Liu, M. Liu, F. Peng, P. An, B. Qin, H. Zhou, H. Li, Z. He. Low-overpotential selective reduction of CO_2 to ethanol on electrodeposited Cu_xAu_y nanowire arrays. *J. Energy Chem.* **2019**, *37*, 176-182.

[14] K. Zhao, Y. Liu, X. Quan, S. Chen, H. Yu. CO_2 electroreduction at low overpotential on oxide-derived Cu/carbons fabricated from metal organic framework. *ACS Appl. Mater. Inter.* **2017**, *9*, 5302-5311.

[15] J. Yuan, M.-P. Yang, W.-Y. Zhi, H. Wang, H. Wang, J.-X. Lu. Efficient electrochemical reduction of CO_2 to ethanol on Cu nanoparticles decorated on N-doped graphene oxide catalysts. *J. CO_2 Util.* **2019**, *33*, 452-460.

[16] Y. Liu, Y. Zhang, K. Cheng, X. Quan, X. Fan, Y. Su, S. Chen, H. Zhao, Y. Zhang, H. Yu, M. R. Hoffmann. Selective electrochemical reduction of carbon dioxide to ethanol on a boron- and nitrogen-co-doped nanodiamond. *Angew. Chem. Int. Ed.* **2017**, *56*, 15607-15611.

[17] Y. Song, S. Wang, W. Chen, S. Li, G. Feng, W. Wei, Y. Sun. Enhanced ethanol production from CO_2 electroreduction at micropores in nitrogen-doped mesoporous carbon. *ChemSusChem* **2019**, *13*, 293-297.

[18] Y. Song, W. Chen, C. Zhao, S. Li, W. Wei, Y. Sun. Metal-free nitrogen-doped mesoporous carbon for electroreduction of CO_2 to ethanol. *Angew. Chem., Int. Ed.* **2017**, *56*, 10840-10844.

[19] Z. Chen, T. Wang, B. Liu, D. Cheng, C. Hu, G. Zhang, W. Zhu, H. Wang, Z.-J. Zhao, J. Gong. Grain-boundary-rich copper for efficient solar-driven electrochemical CO_2 reduction to ethylene and ethanol. *J. Am. Chem. Soc.* **2020**, *142*, 6878-6883.

[20] D. Gao, I. Zegkinoglou, N. J. Divins, F. Scholten, I. Sinev, P. Grosse, B. R. Cuenya. Plasma-activated copper nanocube catalysts for efficient carbon dioxide electroreduction to hydrocarbons and alcohols. *ACS Nano* **2017**, *11*, 4825-4831.

[21] Q. Zhu, X. Sun, D. Yang, J. Ma, X. Kang, L. Zheng, J. Zhang, Z. Wu, B. Han. Carbon dioxide electroreduction to C_2 products over copper-cuprous oxide derived from electrosynthesized copper complex. *Nat. Commun.* **2019**, *10*, 3851.

[22] Y. X. Duan, F.-L. Meng, K.-H. Liu, S.-S. Yi, J.-M. Yan, Q. Jiang. Amorphizing of Cu nanoparticles toward highly efficient and robust electrocatalyst for CO_2

reduction to liquid fuels with high faradaic efficiencies. *Adv. Mater*. **2018**, *30*, 1706194.

[23] J. Yuan, W.-Y. Zhi, L. Liu, M.-P. Yang, H. Wang, J.-X. Lu. Electrochemical reduction of CO_2 at metal-free N-functionalized graphene oxide electrodes. *Electrochim. Acta* **2018**, *282*, 694-701.

[24] A. Dutta, I. Z. Montiel, R. Erni, K. Kiran, M. R. J. Drnec, P. Broekmann. Activation of bimetallic AgCu foam electrocatalysts for ethanol formation from CO_2 by selective Cu oxidation/reduction. *Nano Energy* **2020**, *68*, 104331.
